# Chitooligosaccharides improves intestinal mucosal immunity and intestinal microbiota in blue foxes

**DOI:** 10.3389/fimmu.2024.1506991

**Published:** 2024-11-19

**Authors:** Jiali Wei, Jing Su, Guiwu Wang, Wei Li, Zhengshun Wen, Huitao Liu

**Affiliations:** ^1^ Department of Livestock and Poultry Breeding, Institute of Special Animal and Plant Sciences, Chinese Academy of Agricultural Sciences, Changchun, China; ^2^ Technological Innovation Center for Fur Animal Breeding of Hebei, Shijiazhuang Academy of Agriculture and Forestry Sciences, Shijiazhuang, China; ^3^ Xianghu Laboratory, Hangzhou, China

**Keywords:** chitooligosaccharides, microbiota, intestinal barrier, mucosal immunity, blue fox

## Abstract

**Objective:**

Gut health is critical to the health of the host. This study was conducted to investigate the effects of Chitooligosaccharides (COS) on intestinal morphology, intestinal barrier, intestinal immunity and cecum microbiota of blue foxes.

**Methods:**

Seventy-two 125-day-old blue foxes were randomly divided into basal diet (BD) group, 200 ppm COS1 (1.5 kDa) group and 200 ppm COS2 (3 kDa) group for 8 weeks.

**Results:**

We elucidated that dietary COS1 supplementation promoted the development of intestinal villus morphology in blue foxes. Importantly, COS1 increased the number of goblet cells in duodenum, jejunum and ileum by 27.71%, 23.67%, 14.97% and S-IgA secretion in duodenum, jejunum and ileum by 71.59% and 38.56%, and up-regulate the expression of Occludin and ZO-1 by 50.18% and 148.62%, respectively. Moreover, COS1 promoted the pro-inflammatory and anti-inflammatory balance of small intestinal mucosa, and increased the diversity of cecum microbiota of blue foxes, especially Lactobacillus_agilis and Lactobacillus_murinus, and up-regulated the signaling pathways related to polysaccharide decomposition and utilization.

**Conclusion:**

Here, we present dietary COS1 (1.5 kDa) can promote intestinal villus development, enhance intestinal barrier function, regulate intestinal immune balance and cecum microbiota homeostasis.

## 1 Introduction

The blue fox (*Alopex lagopus*) is a mammal of the Arctic fox genus Canidae of the carnivoran order, with a circumpolar distribution in the northern hemisphere (1). The blue fox fur is soft and luster, with dense plush and straight needle hair (2). The main purpose of breeding blue foxes is to obtain high yield and quality fur for high economic benefits (3). The blue fox is a kind of typical carnivore, which inhabits Arctic islands, mainly feeds on Marine resources such as seabirds, new-born seal pups, and fish and shrimp (4). The intestine of the blue fox is short, about 4.3 times the length of the body (about 235 cm), and the time for food to pass through the gastrointestinal tract is only 20-25 hours (5, 6). Weaning, mother-child separation, and environmental factors can trigger weaning stress in newborn animals, reduce circulating antibody levels, cause microbiota imbalance, change intestinal morphology and function, and weaken digestion and absorption capacity (7). The growing period (125 days of age) is the key period of post-weaning development of blue foxes. The health status of blue fox is a key determinant of fur quality.

Chitosan oligosaccharides (COS) are co-oligomers of N-acetylglucosamine and D-glucosamine linked by *β* - (1→4) glucoside bonds (8). COS is usually prepared by hydrolysis or deacetylation of chitin/chitosan, the primary source is abundant marine biological resources, such as shrimp shells and crab shells (the second most abundant polysaccharide in nature) (9). The mean molecular weight (MWs) of COS is less than 3.9 kDa and the degree of polymerization is between 2-20 (10). COS has the characteristics of high degree of deacetylation, high water solubility, high biocompatibility and low viscosity (11), which endows it with significant antibacterial activity, immune activity, probiotic activity and lipid lowering activity (12–15). Moreover, it showed to protect the intestinal barrier and regulate the intestinal flora (16). In mouse models of colitis and IPEC-J2 cells, COS was found to directly enhance intestinal barrier function, and COS intervention also promoted the richness and diversity of intestinal flora. It was confirmed that COS-modified fecal microbial transplantation can enhance intestinal barrier function (16). In mice, one *in vivo* study demonstrates that COS (200 mg/kg-1) treatment promotes the population of Bacteroidetes, but inhibits the Proteobacteria phylum. At the genus level, COS treatment reduces the population of probiotic Lactobacillus, Bifidobacterium, and harmful Desulfovibrio bacteria (17). Meanwhile, *in vitro* fermentation assessments conducted in the same study show that COS decreases the number of Escherichia/Shigella pathogens (17). In pigs, dietary supplementation of COS (Mw =1.5 kDa) increases the number of Bifidobacteria and Lactobacilli (18).Therefore, COS is widely used in livestock and poultry production as a natural growth promoter, immunomodulator and potential prebiotic (19).

In this study, we selected COS as a potential prebiotic as a feed additive to explore its effects on intestinal morphology, intestinal barrier, intestinal immunity and cecum microbiota of blue foxes. We hope to provide scientific data for marine-derived oligosaccharides regulating gut microbiota and gut health in carnivores.

## 2 Materials and methods

### 2.1 Source of materials

COS1, chitooligosaccharides (molecular weight 1.5 kDa), and COS2, chitooligosaccharides (molecular weight 3 kDa) were purchased from Golden-Shell Pharmaceutical Co., Ltd. (Zhejiang, China). All animal-specific procedures were approved and authorized by the Chinese Academy of Agricultural Sciences Animal Care and Use Committee, and the Institute of Special Animal and Plant Sciences Wild Animal and Plant Subcommittee (ISAPSWAPS2016000302).

### 2.2 Animals and experimental design

A total of 72 125-day-old blue foxes were randomly assigned into 3 dietary groups supplemented with nothing, COS1 or COS2 at 200 ppm. The composition and nutrient levels of the basal diet are shown in **Table 1**. The addition concentration of COS was set according to the previous literature (20). Each group contained 3 replicates with 8 foxes per replicate (half male and half female). The adaptation period was 7 days, and the formal experiment lasted for 60 days. Each blue fox was housed in a single cage with a size of 110 cm × 90 cm × 90 cm. Foxes were fed daily at 8:00 and 16:00 and had water *ad libitum*. Feeding experiments were conducted outdoors under natural light. All foxes remained in good health during the whole feeding period. In experiment 1 and 60 days respectively weighing blue fox weight on an empty stomach in the morning, used to measure the average daily gain (ADG) of blue fox.

**Table 1:** Basal diet composition and nutrient level of blue fox during growing period (%, as-fed basis).

Ingredients (%)		Nutrient composition	
Corn	35.50	ME (MJ/kg)	12.97
Soybean meal	16.40	CP (%)	29.08
Fish meal	17.50	EE (%)	12.40
Meat and bone meal	5.00	Lys (%)	2.28
Chellocken meal	5.00	Met (%)	1.23
Corn gluten meal	5.00	Ca (%)	2.40
Corn germ meal	4.00	P (%)	1.18
Fish oil	3.00		
Lard	4.50		
NaCl	0.10		
Premix^1^	4.00		
Total (%)	100.00		

ME, metabolic energy; CP, crude protein; EE, ether extract; Lys, lysine; Met, methionine; Ca, calcium; P, phosphorus.

^1^ Premix provided per kilogram of diet: vitamin A, 200,000 IU; vitamin D_3_, 40,000 IU; vitamin E, 2,500 IU; vitamin B1, 125 mg; vitamin B2, 200 mg; vitamin B3, 500 mg; vitamin B6, 200 mg, vitamin B12, 2.5 mg; vitamin K3, 40 mg; pantothenic acid, 800 mg; biotin, 7.5 mg; folic acid, 100 mg; vitamin C,7,500 mg;Fe, 2000 mg; Zn, 1500 mg; Cu, 500 mg; Mn, 400 mg; I, 15 mg; Co, 7.5 mg; Se, 5 mg.

### 2.3 Sample collection

The slaughter test was carried out on day 60 of the feeding experiment, and the test animals were fasted and had free access to water for 12 h before sacrifice. Nine blue foxes (3 in each replicate) were randomly selected, blood samples were collected from the hind limb vein, serum was isolated from blood samples after centrifugation and stored at -20°C for the detection of cytokines content. Blue foxes were killed by injection of 5 mL of succinic acetylcholine, skinned and dissected. The tissues of duodenum, jejunum and ileum were collected for histological examination. The mucosa of the duodenum, jejunum, and ileum and cecum contents were quickly frozen in liquid nitrogen and stored at -80°C for subsequent detection of relative quantification of genes and detection of microbiota diversity.

### 2.4 Histological examination

Duodenum, jejunum and ileum tissue of 5 blue foxes from each group were fixed with 4% paraformaldehyde and Carnoy’s Fluid, embedded in paraffin. Serial sections (5 to 8 *μ*m) for hematoxylin - eosin (H&E) and Alcian staining. Images were acquired through the Lycra microscope. Villi height is measured from the tip of the villi to the junction of the recess (*μ*m). Villus width is defined as the width at the widest part of the villus (*μ*m). Crypt depth was defined as the depth of adjacent villi invagination (*μ*m). Intestinal morphology and goblet cell number were determined by measuring at least 6 different areas in each section using the Image-Pro-Plus Image analysis system.

### 2.5 RNA isolation and real-time quantitative PCR in jejunum mucosa

The RNA was extracted using the RNAiso kit and assayed for purity and concentration. The cDNA was synthesized using a reverse transcription kit (PrimeScript ™ PT Master Mix). Real-time PCR for measuring gene expression was conducted using TB Green Premix Ex Taq ™ kit. Gene-specific primer sequences for Occludin, zonula occludens-1 (ZO-1), interleukins-10 (IL-10), transforming growth factor-*β*1 (TGF-*β*1) and tumor necrosis factor-*α* (TNF-*α*) are list in **Table 2**. The cycling conditions used to amplify cDNA were as follows: 95°C for 5 min; 40 cycles for 95°C for 10 s, 60°C for 35 s. The relative gene expression levels were calculated using the 2^-^ *^δδ^* ^Ct^ method.

**Table 2:** Primer sequences for qPCR.

Gene	Forward primers (from 5’ to 3’)	Reward primers (from 5’to 3’)
*β-Actin*	ACCCACACGGTGCCCATC	CTTGATGTCACGCACGATTTCC
*Occludin*	ATCCAACTGCCCAGGCTTCT	ATCACCATGAACCCCAGGACA
*Zo-1*	CATAACAGATACAGACCAGAAGCACAG	AGGAGGGACAACCGCAGCAC
*IL-10*	CTGGACAACATACTGCTGACCG	CTTGATGTCTGGGTCGTGGTTC
*TGF-β1*	AGTGCCTGAGCCTGTCTTGC	CCAGTGACATCAAAGGACAGCC
*IL-1β*	CAAATACCTGGTGCTGTCTAACTCG	GGGCTTCCCATCCTTCATCA
*TNF-α*	ATGTTGTAGCAAACCCCGAAGC	CAAAGCGGCTGATGGTGTGG

*ZO-1*, Zonula occludens-1; IL-10, Interleukin-10; TGF-*β*1, transforming growth factor-β; IL-1*β*, Interleukin-1*β*; TNF-*α*, Tumor necrosis factor-*α*.

### 2.6 Enzyme-linked immunosorbent assay (ELISA)

Enzyme-linked immunosorbent assay kits were used to detect the levels of IgG, IgA, IgM, IL-2, IL-6, IL-1*β*, TNF-*α*, IL-10, DAO and D-lactate in serum and jejunal mucosa, and the content of S-IgA in duodenum, jejunum and ileum mucosa. The lower limit of detection was: IgG (2.01 mg/g), IgA (408.37 pg/g), IgM (326.77 *μ*g/g), IL-2 (49.81 pg/g), IL-6 (11.71 pg/g), IL-1*β* (216.04 pg/g), TNF-*α* (18.48 pg/g), IL-10 (14.30 pg/g), DAO (27.07 pg/g), D-lactate (0.79 mmol/L) and S-IgA (98.10 pg/g). Fox Immunoglobulin G (IgG) kit (No. 01.01.0143.10075.00), Fox Immunoglobulin A (IgA) kit (No. 01.01.0143.10076.00), Fox Immunoglobulin M (IgM) kit (No. 01.01.0143.10077.00) Fox interleukin-2 (IL-2) kit (No. 01.01.0143.10078.00), Fox interleukin-4 (IL-4) kit (No. 01.01.0143.10079.00), Fox interleukin 1β (IL-1β) kit (No. 01.01.0143.10081.00), Fox tumor necrosis factor α (TNF-α) kit (No. 01.01.0143.10082.00), Fox interleukin10 (IL-10) kit (No. 01.01.0143.10080.00), Fox diamine oxidase (DAO) kit (No. 01.01.0143.10083.00), Fox lactic acid (LD (kit) (No. 01.02.0153.10002.00). The above kits were purchased from Shanghai Guchen Biotechnology Co., LTD. Secretory immunoglobulin A(S-IgA) (No. H108-2) was purchased from Nanjing Jiancheng Biotechnology Co., LTD.

### 2.7 Sequencing of cecum microbiota

DNA extraction and sequencing were performed by Nuohezhiyuan Technology Co., LTD., Beijing, China. 16S rRNA genes of distinct regions (16SV4) were amplified used specific primer (515F-806R) with the barcode. Sequencing libraries were generated using NEB Next^®^ Ultra™ II FS DNA PCR- free Library Prep Kit. Quality filtering on the raw tags were performed using the fastq software to obtain high-quality Clean Tags. Species annotation was performed using QIIME2 software. The row reads were deposited into NCBI Sequence Read Archive (SRA) database (Accession Number: PRJNA1066055). The Nuohezhiyuan cloud platform was used for bioinformatics analysis (https://magic.novogene.com).

### 2.8 Statistical analysis

The experimental data were analyzed using one-way ANOVA and differences were compared using Ducan’s Multiple Range Test in SPSS 26.0 software, and expressed as mean ± standard deviation (SD). The statistical significance is defined as *P* < 0.05. Graph pad Prism 8 was used to plot bar chart.

## 3 Results

### 3.1 COS had no adverse effect on the body weight of blue foxes

The effect of dietary supplement COS on body weight of blue foxes are showed in **Table 3**. During the 8-week feeding trial, there were no significant differences in initial body weight, final body weight and average daily gain (ADG) in COS supplement group compared with the BD group (*P* > 0.05). The results showed that supplementation of 200 mg/kg COS1 and COS2 had no adverse effects on body weight of blue foxes during growing period.

**Table 3:** Effects of chitosan oligosaccharide on growth performance of blue fox^1^.

Items	BD	COS1	COS2	*P*-Value
Initial body weight/kg	6.39 ± 0.56	6.35 ± 0.60	6.35 ± 0.29	0.969
Final body weight/kg	12.11 ± 0.50	12.26 ± 0.69	12.04 ± 0.33	0.692
Average daily gain/(g/d)	95.42 ± 2.14^ab^	98,54 ± 3.50^a^	96.39 ± 2.94^b^	0.031

BD, basal diet group; COS1, basal diet supplemented with 200 ppm chitooligosaccharides (molecular weight 1.5 kDa) group. COS2, basal diet supplemented with 200 ppm chitooligosaccharides (molecular weight 3 kDa) group.

^1^ Performance data represent the mean values ± standard deviation of 24 blue fox.

^a,b^ Datas shows as mean values ± sd in the same row with different letters differ at *P* < 0.05.

### 3.2 COS1 increased the development of intestinal villus morphology in blue foxes

**Figure 1:**
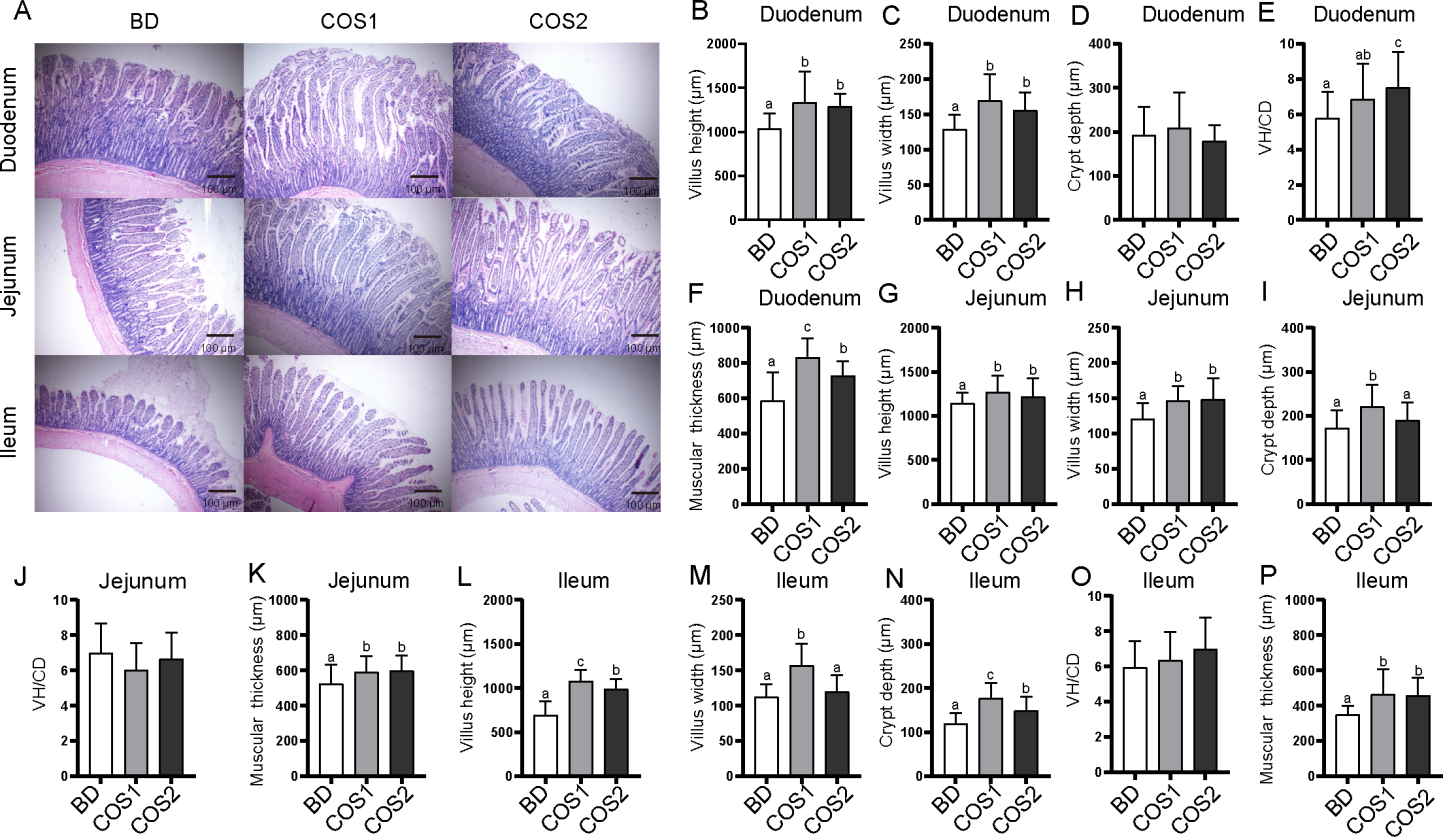
Effects of dietary COS supplementation on morphology and development of intestinal villi of blue fox during growing period. Histological examination of duodenum, jejunum, and ileum stained with H*&*E in BD group, COS1 group (1.5 kDa), COS2 group (3 kDa) (Light microscopy 400×, n = 5) **(A)**. Villus height (*μ*m), villus width (*μ*m), crypt depth (*μ*m), villus height/crypt depth, muscular thickness (*μ*m) in duodenum **(B–F)**, jejunum **(G–K)**, and ileum **(L–P)**. The letters a, b, and c in the bar chart represent significant differences between groups at *P* < 0.05.

In the duodenum, compared with the BD group, the villus height, villus width and muscular thickness in COS1 group increased the most (42.06%, 31.19% and 28.83%, respectively) (*P* < 0.05) (**Figures 1A–C, F**), while the VH/CD ratio increased by 30.3% in the COS2 supplement group (*P* < 0.05), suggesting a risk of hindering villi growth (**Figures 1A, E**). In the jejunum, compared with the BD group, the villus height was increased by 10.83% in the COS1 group, and the villus width and muscular thickness were increased in the COS1 and COS2 groups (*P* < 0.05) (**Figures 1A, G, H, K**). COS1 supplementation showed the highest increase incrypt depth of jejunum, and villus height, villus width, crypt depth, and muscular thickness in the ileum (*P* < 0.05) (**Figures 1A, I, L–N, P**). COS had no significant effects on duodenal crypt depth and VH/CD values of jejunum and ileum (*P* > 0.05) (**Figures 1D, J, O**). These results indicated that supplementation of 200 mg/kg COS1 was beneficial to stimulate the villus morphology and development of ileum in growing blue foxes.

### 3.3 COS improved intestinal barrier function parameters of blue foxes

**Figure 2:**
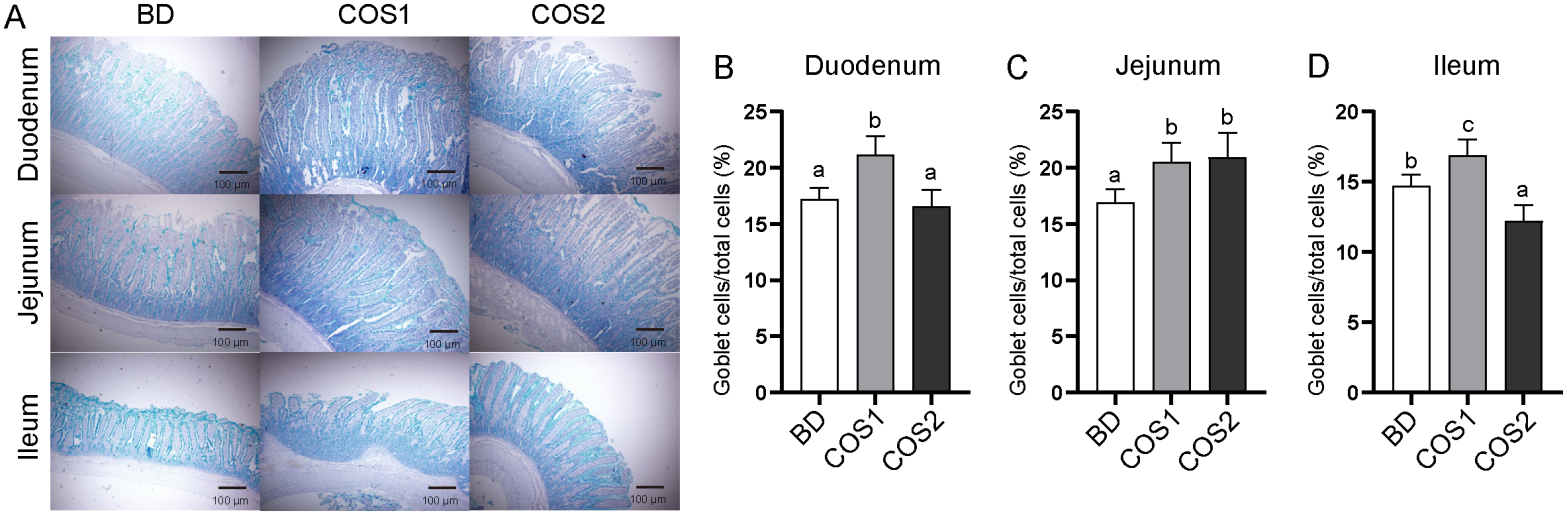
Effect of dietary COS supplementation on the number of small intestinal goblet cells in blue foxes during growing period. Representative images of Alcian staining in duodenum, jejunum, and ileum tissue to observe goblet cells (Light microscopy 400×, n = 5) **(A)**. Proportion of goblet cells in duodenum **(B)**, jejunum **(C)**, ileum **(D)**. The letters a, b, and c in the bar chart represent significant difference between groups at *P* < 0.05.

**Figure 3:**
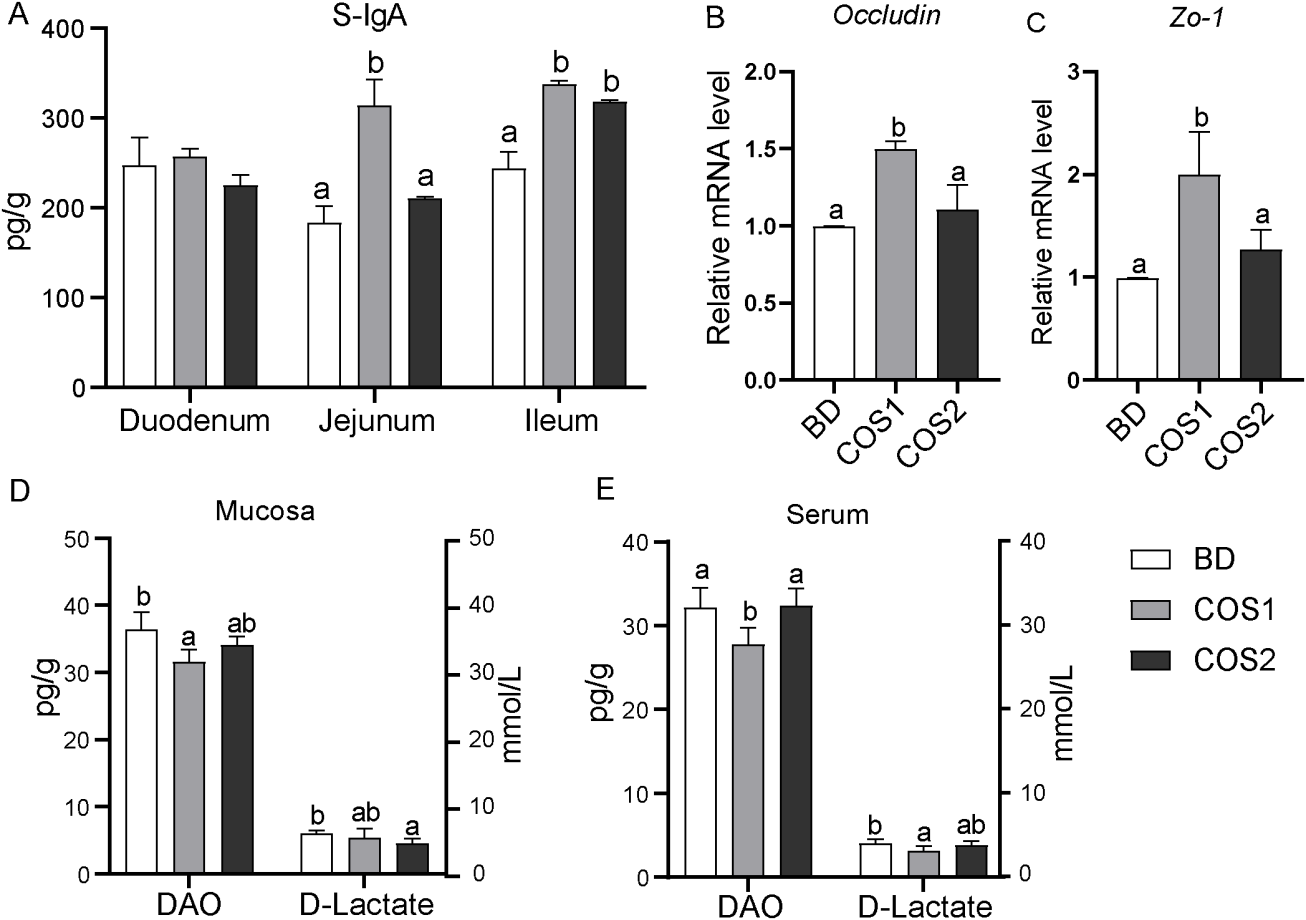
Effects of dietary COS supplementation on intestinal barrier indexes of blue foxes during growing period. Content of secretory immunoglobulin A in duodenum, jejunum, and ileum **(A)**. The relative mRNA expression levels of *Occludin* and zonula occludens 1 (*ZO-1*) in small intestinal mucosa were detected by RT-qPCR **(B, C)**. Levels of diamine oxidase and D-lactic acid in intestinal mucosa **(D)** and serum **(E)**. The letters a, b, and c in the bar chart represent significant differences between groups at *P* < 0.05.

Compared with BD group, the number of goblet cells in duodenum, jejunum and ileum in COS1 group increased by 27.71%, 23.67% and 14.97%, respectively (*P* < 0.05) (**Figures 2A–D**). Meanwhile, the S-IgA content in jejunum and ileum mucosa of COS1 group was increased by 71.59% and 38.56% compared with BD group, respectively (*P* < 0.05) (**Figure 3A**). Moreover, the jejunal mucosa of blue foxes in COS1 group showed up-regulated *Occludin* and *ZO-1* mRNA level. (*P* < 0.05) (**Figures 3B, C**). In the jejunal mucosa and serum, the levels of DAO and D-Lactate in COS1 group all decreased, compared with those in BD group (*P* < 0.05) (**Figures 3D, E**). These results suggested that COS1 supplementation has the potential to enhance intestinal barrier function in growing blue foxes.

### 3.4 COS promoted intestinal immune balance in blue foxes

**Figure 4:**
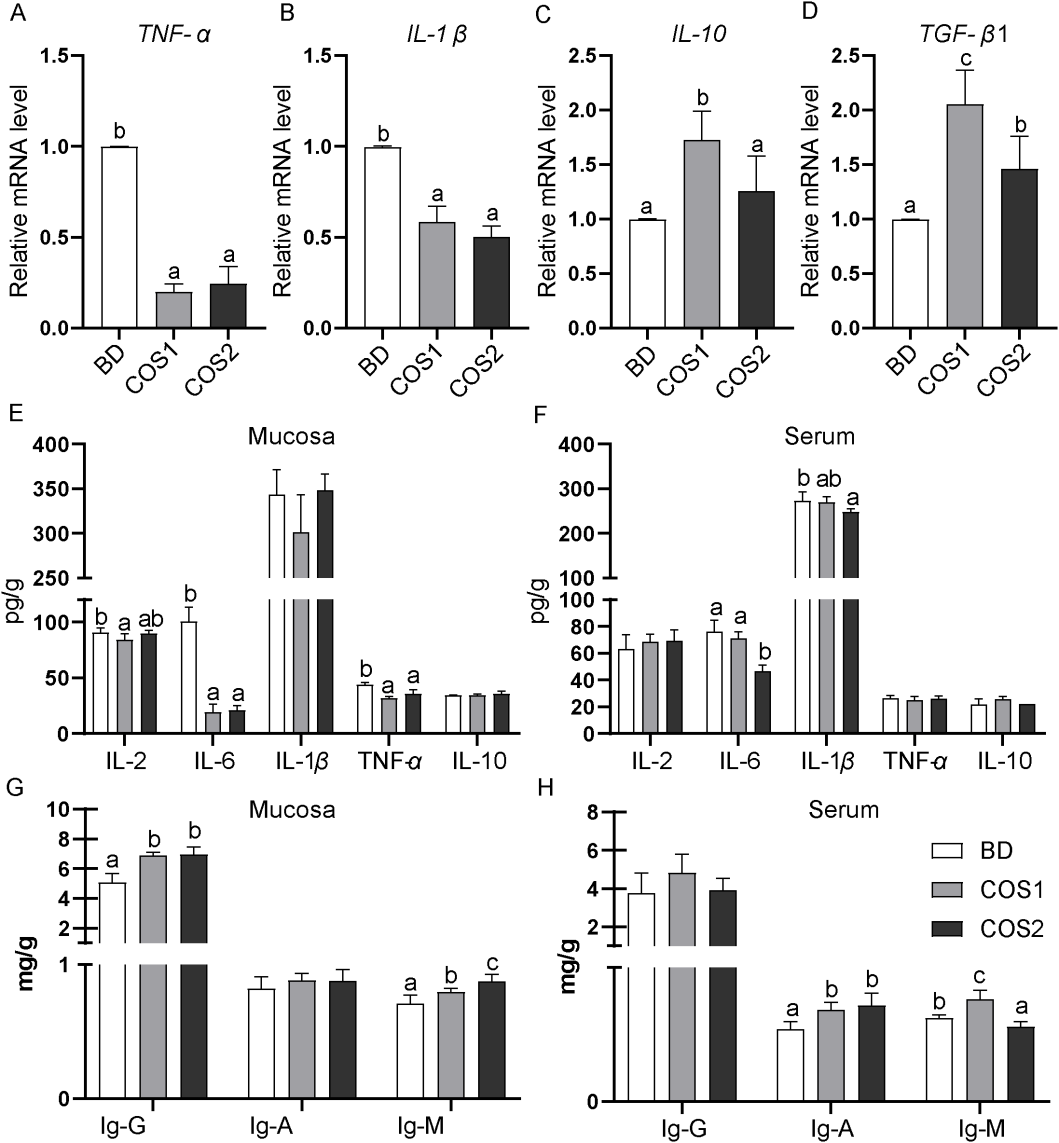
Effects of dietary COS supplementation on intestinal immunity of blue foxes during growing period. The relative mRNA expression levels of genes for inflammatory cytokines in small intestinal mucosa were detected by RT-qPCR **(A–D)**. Levels of inflammatory cytokines in intestinal mucosa and serum **(E, F)**. Contents of immunoglobulin-G, immunoglobulin-A, and immunoglobulin-M in intestinal mucosa and serum **(G, H)**. The letters a, b, and c in the bar chart represent significant differences between groups at *P* < 0.05.

In the jejunal mucosa, compared with the BD group, the mRNA levels of pro-inflammatory cytokines *TNF-α* and *IL-1β* in the COS1 and COS2 supplementation groups were decreased, and *TNF-α* in the COS1 group decreased the most by 79.80% (*P* < 0.05) (**Figures 4A, B**). Compared with the BD group, the mRNA levels of anti-inflammatory factors *IL-10* and *TGF-β1* in COS1 supplementation group increased the most, which were 72.66% and 106.32%, respectively (*P* < 0.05) (**Figures 4C, D**). In the jejunal mucosa, the contents of pro-inflammatory cytokines IL-2, IL-6 and TNF-*α* were the lowest in COS1 supplementation group, especially the content of IL-6 was decreased by 81.03% compared with BD group (*P* < 0.05) (**Figure 4E**). In serum, the concentrations of proinflammatory cytokines IL-6 and IL-1*β* were lower in the COS2 group than in the BD group (*P* < 0.05) (**Figure 4F**). Antibodies IgG, IgA and IgM play an important bridging role in the immune response (21). Compared with the BD group, the jejunum mucosa of IgG and IgM concentration in COS1 and COS2 supplementary groups in are increased, the serum IgA and IgM in COS1 supplementary group content increased (*P* < 0.05) (**Figures 4G, H**). These results proved that COS1 supplementation could promote the dynamic balance of intestinal mucosal immunity in blue foxes.

### 3.5 COS modulated cecum microbiota of blue foxes

**Figure 5:**
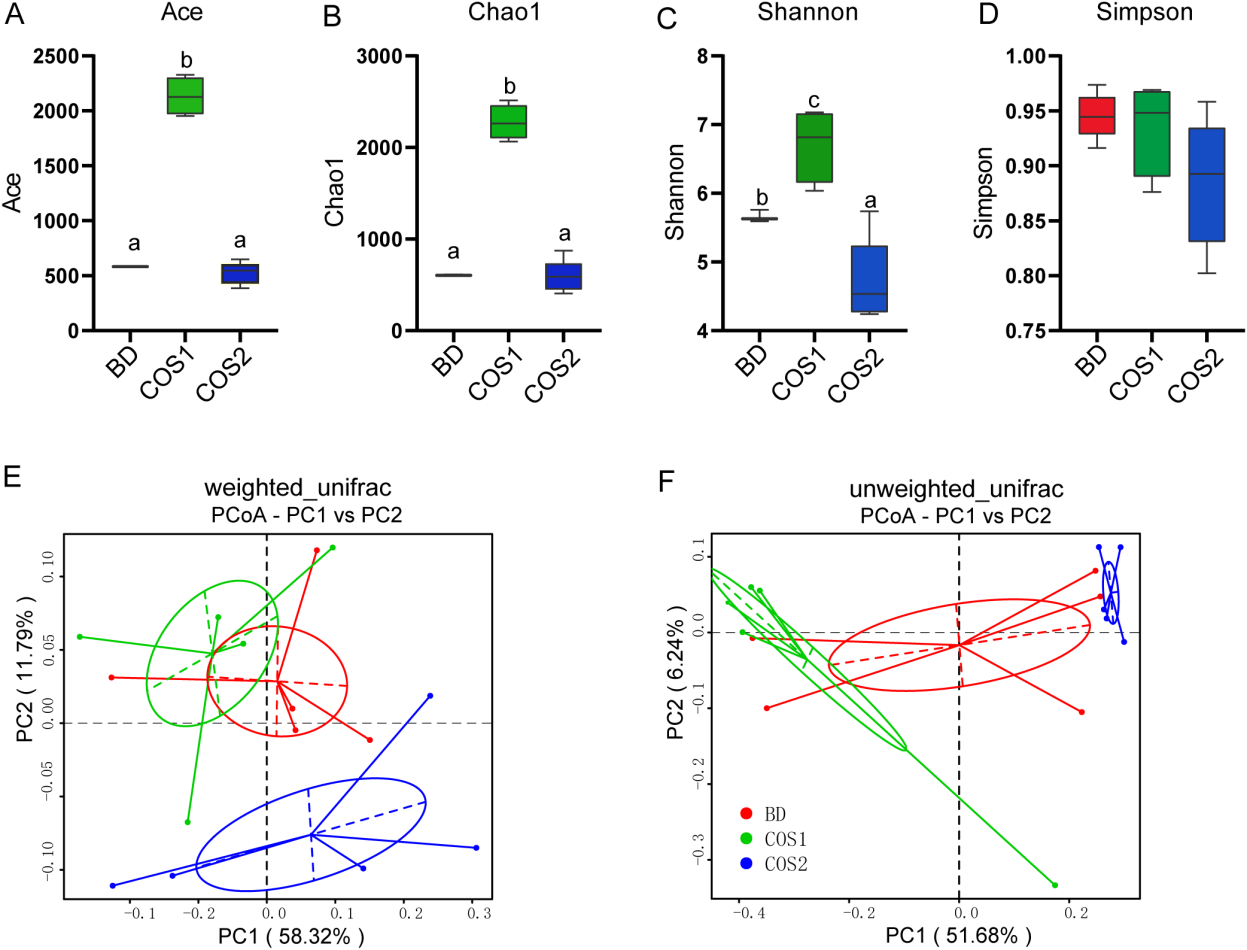
Effects of dietary COS supplementation on cecal microflora diversity in growing blue foxes. Abundance index: Ace Index **(A)** and Chaos Index **(B)**. Diversity index: Shannon index **(C)** and Simpson index **(D)**. Principal Coordinate Analysis (PCoA) analyzed community structure diversity: weighted_unifrac **(E)** and unweighted_unifrac **(F)**. The letters a, b, and c in the bar chart represent significant differences between groups at *P* < 0.05.

Compared with the BD group, the Ace, Chao and Shannon indexes of the COS1 supplementation group were significantly increased by 266.82%, 275.06% and 20.17% (*P* < 0.05) (**Figures 5A–D**), indicating that COS1 supplementation increased the diversity of intestinal groups of blue foxes. The weighted_unifrac distance considered the effects of microbial species abundance and species on community structure composition, while the unweighted_unifrac distance only considered the effects of species. In both distance algorithms, the sample points in the COS1 group and the COS2 group are clustered separately (**Figures 5E, F**), indicating that there are differences in the species and abundance of microorganisms between the two groups.

**Figure 6:**
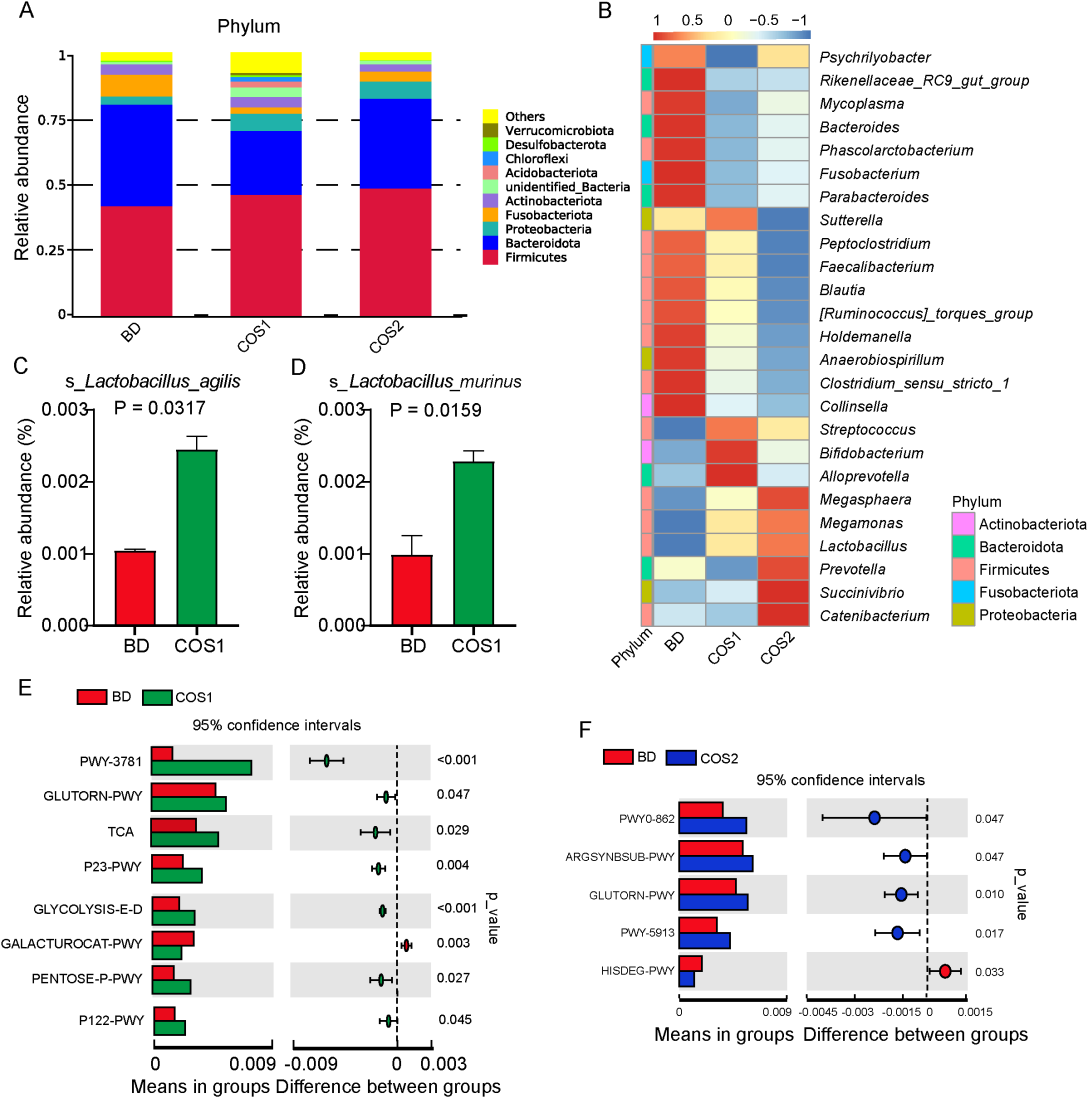
Effects of dietary COS supplementation on cecal microflora composition of blue foxes during growing period. Relative abundance histogram at the Phylum level **(A)**. Relative abundance chart at the Genus level **(B)**. The column of *Lactobacillus* with significant difference at Species level (*P* < 0.05) **(C, D)**. Differential enrichment analysis of microbial gene function pathway prediction: BD vs COS1 **(E)**, and BD vs COS2 **(F)**.

At the phylum level, the gut microbiota of blue fox is mainly composed of Firmicutes and Bacteroidetes (**Figure 6A**). Fusobacteria and Phascolarctobacterium were enriched in BD group, while reduced in COS1 and COS2 group (**Figures 6A, B**). Compared with BD and COS2 group, Acidobacteriota, Chloroflexi, and Verrucomicrobiota in COS1 group were more abundant (**Supplementary Figures S1**, **S2**). At the genus level, COS1 supplementation resulted in enrichment of *Alloprevotella* classified in the phylum Bacteroides, *Bifidobacterium* classified in the phylum Actinobacteria and *Lactobacillus* classified in the phylum Firmicutes (**Figure 6B**). COS2 supplementation resulted in enrichment of *Prevotella* classified in the phylum Bacteroides, and *Catenibacterium*, *Megamonas*, and *Megasphaera* classified in the phylum Firmicutes (**Figure 6B**). In order to reveal which lactobacillus is responsive to COS1, the enrichment of *Lactobacillus* at species level in each group was further explored. The datas showed that compared with BD group, only the relative abundance of *Lactobacillus*_*agilis* and *Lactobacillus*_*murinus* in COS1 group increased by 130.84% and 134.45% (*P* < 0.05) (**Figures 6C, D**).

In the prediction of microbial gene function, the signaling pathway of L-ornithine biosynthesis (GLUTORN-PWY) was up-regulated in both COS1 and COS2 compared with BD group (*P* < 0.05) (**Figures 6E, F**). Interestingly, compared to the BD group, In COS1 group, signaling pathways such as glycolysis and Entner-Doudoroff pathway (GLYCOLYSIS-E-D), D-galacturonate degradation I pathway (GALACTUROCAT-PWY), pentose phosphate pathway (PENTOSE P-PWY), and heterolactic fermentation pathway (P122-PWY) were upregulated (*P* < 0.05) (**Figure 6E**). These up-regulated pathways are related to the decomposition and utilization of polysaccharides.

### 3.6 Correlation between intestinal barrier factors, intestinal immune parameters, and differential bacteria

**Figure 7:**
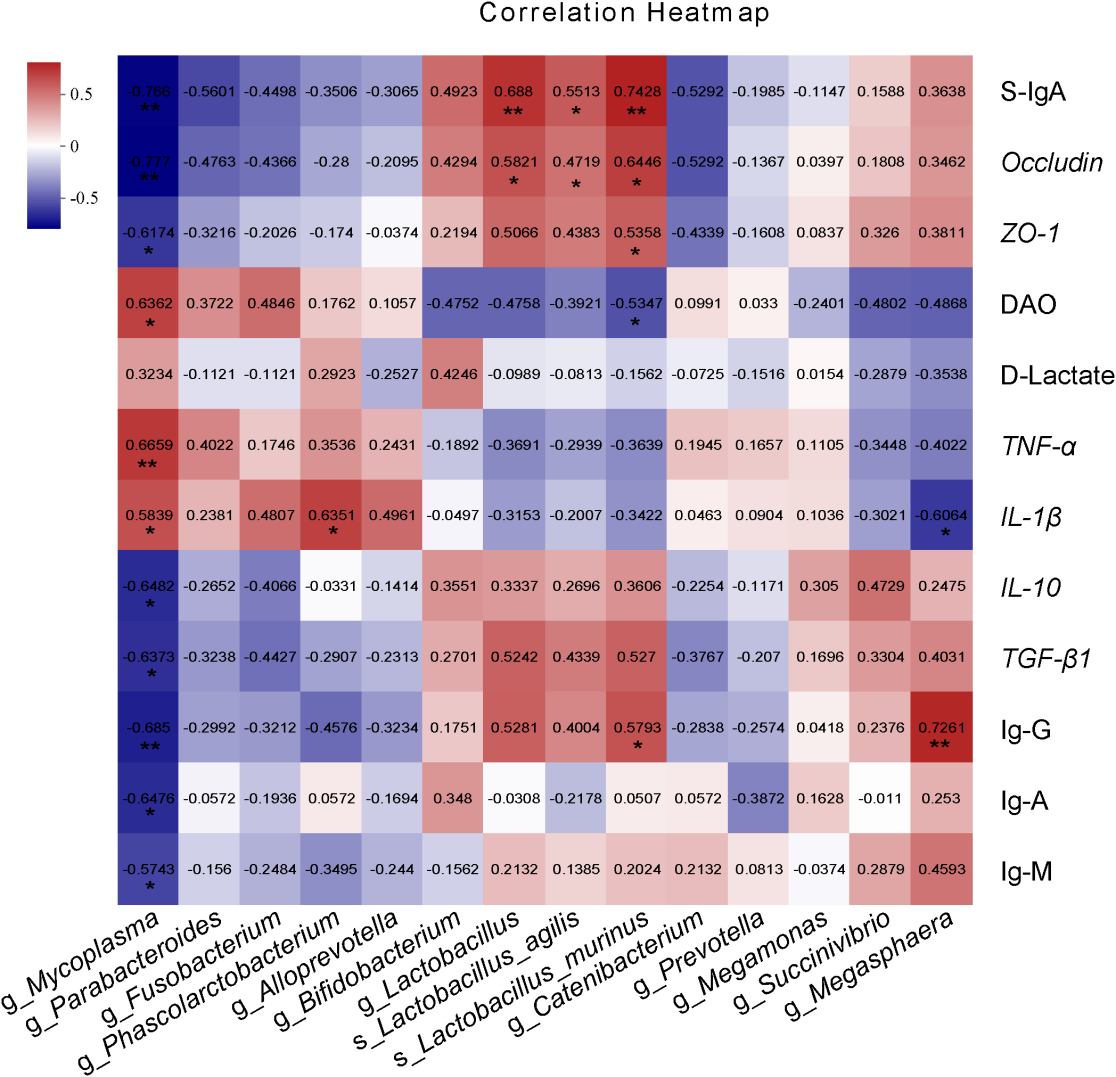
Spearman correlation analysis of intestinal barrier factors and intestinal immune parameters with differentially enriched cecum microbiota. The red grid indicates a positive correlation; The blue grid shows the negative correlation. The numbers in the grid represent the correlation coefficient. The “*” “**”represent significant differences between groups at *P* < 0.05, *P* < 0.01.

**Figure 7** shows the Spearman correlation analysis between intestinal barrier factors, intestinal immune parameters and differentially enriched bacteria in cecum. Intestinal barrier factors S-IgA, *Occludin*, and *ZO-1* showed strong positive correlation with enriched bacteria in COS1 group (especially *Lactobacillus_agilis* and *Lactobacillus*_*murinus*) (*R* > 0.4, *P* < 0.05), and weak positive correlation with enriched bacteria in COS2 group (*R* > 0.1, *P* < 0.05). The DAO was strongly positively correlated with the enriched *Mycoplasma*, *Parabacteroides* and *Fusobacterium* in group BD (*R >*0.4, *P* < 0.05), and was negatively correlated with enriched bacteria in COS1 group and enriched Succivibrio and Megasphaera in COS2 group (*R* < -0.3, *P* < 0.05). The correlation between pro-inflammatory factors *TNF-α* and *IL-1β* and intestinal bacteria was similar to DAO (*P* < 0.05). The correlation between anti-inflammatory factors *IL-10* and *TGF-β1* and intestinal bacteria was similar to intestinal barrier factors (*P* < 0.05). Intestinal immunoglobulin Ig-G was strongly positively correlated with *Lactobacillus*, *Lactobacillus_murinus* and *Megasphaera* (*R* > 0.4, *P* < 0.05). To sum up, the correlation evaluation of COS1 is better than that of COS2. Therefore, we speculate that COS1 may specifically enrich beneficial bacteria, especially *Lactobacillus_agilis* and *Lactobacillus_murinus*, to protect the intestinal barrier and regulate the intestinal immune function.

## 4 Discussions

Host intestinal health is closely related to intestinal mucosal immunity and intestinal microecology. Chitooligosaccharides (COS) as potential prebiotics can improve the balance of intestinal flora and mediate intestinal immunity (22). However, studies on COS in intestinal immunity and microbial regulation of blue foxes are still limited. Hence, in the present study, diets containing two molecular weights of COS1 (1.5 kDa) and COS2 (3 kDa) were fed to 125-day-old blue foxes for 8 weeks. This study found that short-term feeding COS had no adverse effect on the body weight of blue foxes. Compared with COS2, COS1 has a better effect on promoting intestinal villi development, intestinal mucosal immunity, maintaining inflammatory homeostasis and regulating intestinal microecology. These results demonstrate that COS1 can regulates intestinal mucosal immunity and intestinal microbiota of blue foxes to promoted intestinal health.

The blue fox is an important fur economic animal, and the healthy blue fox shows bright fur plate and dense texture (23). In this study, COS had no effect on the body weight of blue foxes. However, during our experiment, we observed that COS-supplemented blue foxes had smoother and lustrous hair. Whether COS supplementation promotes growth performance in pigs at different growth stages varies depending on the dose, molecular weight of COS, and duration of supplementation (24–26). These results indicated that COS may promote the absorption of nutrients in the fur development of blue foxes, rather than reflected in the weight gain.

COS increased the morphological characteristics of small intestinal villus. The morphological integrity of villi greatly influences the digestion and absorption capacity of the small intestine (27). The morphological characteristics include villus height, villus width, crypt depth and muscular thickness, which mapped nutrient absorption, intestinal barrier function and immunity (28–30). In this study, dietary COS supplementation had the greatest effect on intestinal villi height, villi width and muscular thickness. Consist with our results, Ruixia Lan and Youssef, Islam M. reported that COS increased the ileal villus height and decreased the crypt depth in broilers and laying hens, respectively (31, 32). Similar results were also reported by Dingfu Xiao, who indicated that COS enhanced the villus height/crypt depth in weaned piglets (33). Our results further explore a role of COS in stimulating the morphology of the ileum of blue foxes during growing period.

COS1 had positive effects on mucosal and epithelial barrier function of blue foxes. Goblet cells continuously secrete mucus into the intestinal lumen to remove and block contact with harmful bacteria (34). S-IgA distributed in the mucous layer interferes with pathogen adherence or penetration of the mucosal barrier through antibody-dependent specific immunity and glycan-dependent innate immunity (35). In this study, COS1 supplementation increased the number of small intestinal goblet cells and S-IgA content in jejunum and ileum mucosa. These results are consistent with findings for other species, which supplementation COS (1-2.2 kDa) can increase the goblet cells number of piglets and activate the secretory pathway of S-IgA and enhance the intestinal mucosal immunity of mice (33, 36). Tight junctions are important factors for the integrity of the epithelial barrier, and Occludin and ZO-1 are key proteins in intestinal tight junctions (37). Our results was consistent with a previous finding that dietary COS1 supplementation increased the expression of tight junction in the jejunum of LPS-challenged mice (38).D-lactic acid is a byproduct of bacterial metabolism and occurs only in human micromolar concentrations under normal conditions (39). Diamine oxidase (DAO) is an intracellular enzyme at the tip of the intestinal villi in mammals (40). When the intestinal mucosal barrier is damaged, the release of both is increased and enters the bloodstream. In this study, we demonstrated that COS1 supplementation reduced DAO and D-LA levels, suggesting that COS1 can reduce intestinal permeability in blue foxes. Taken together, these results indicated that COS1 improved the intestinal barrier function in growing blue foxes.

COS regulated the homeostasis of inflammation in blue foxes. Lipid overload associated with obesity induces low-grade tissue inflammation in the body (41). The blue fox belongs to the carnivorous canid family, and the fat in its diet is its main source of energy, and the fat supply in the growing period is about 25-40% (42). Previous studies have shown that COS supplementation alleviated chronic inflammation induced by LPS (43). Moreover, dietary COS supplementation significantly reduced the mRNA expression levels and cytokine contents of inflammatory factors TNF-*α*, IL-*β* and IL-6 in host tissues (44, 45). Consistent with the results of previous studies, in this study, we also found that COS can reduce the level of pro-inflammatory factors in the intestinal mucosa of blue foxes (46), and increased the level of anti-inflammatory factors (such as IL-10), so as to maintain the dynamic balance of intestinal immunity of blue foxes (47). IgG, IgA and IgM can cooperate with each other to mediate intestinal immune adaptation and immune defense against pathogens, and contribute to intestinal homeostasis (48). In this study, COS1 and COS2 supplementations both increased the immunoglobulin levels. These results indicate that COS may regulate intestinal immunity in growing blue foxes by regulating inflammatory balance and immunoglobulin levels.

The rich diversity of intestinal flora lead to functional redundancy, which could maintain the normal operation of intestinal microbiota when the external environment is disturbed (49). In this study, COS1 supplementation increased the richness index and diversity index of intestinal bacteria in growing blue foxes, which was consistent with Huang et al. ‘s study in mice (50). In addition, COS1 and COS2 supplementation both reduced the abundance of Fusobacteria and Phascolarctobacterium. Fusobacteria was intestinal commensal bacteria and opportunistic pathogens, and their redundancy was associated with intestinal infections (51). Further studies have found that *Fusobacterium nucleatum* secretes antagonistic substances to repel probiotics (such as *Bifidobacterium*), resulting in microbiota dysbiosis (52). Phascolarctobacterium was accompanied by the development of obesity in the dog (53), moreover, in obese mice models, it was strongly positively correlated with TC and LDL-C in serum and TG and MDA in liver (54). In this study, the supplementation of COS1 enriched the abundance of Acidobacteriota, Chloroflexi, and Verrucomicrobiota. Acidobacteriota can effectively degrade cellulose, hemicellulose and xylan in nature (55). Verrucomicrobia is an abundant and highly specialized degrader of fucosan and other complex polysaccharides (56). In this study, COS1 enriched *Alloprevotella*, *Bifidobacterium* and *Lactobacillus*. Alloprevotella classified to Bacteroides, are typical dietary fiber degrading bacteria (57). This is consistent with the enrichment of glycolysis and Entner-Doudoroff pathway (GLYCOLYSIS-E-D), D-galacturonate degradation I pathway (GALACTUROCAT-PWY), and pentose phosphate pathway (PENTOSE P-PWY) in microbial function prediction after COS1 supplementation in this study. The enrichment of *Bifidobacterium* could regulate the expression of mucin proteins and immune homeostasis (58, 59). Moreover, *Bifidobacterium* can adhere to dietary glycans such as chitin (60), and dehydrolyze oligosaccharides by expressing a variety of extracellular and cytosolic glycosyl hydrolases (GHs) (61). COS can stimulate the cell density of Lactobacillus more than 4 times, and the mode of promoting the growth of lactobacillus was the same as that of cellobiose (62, 63). These data indicate that COS1 supplementation in the blue fox diet during the growing period may play a role in regulating the intestinal microecological balance through the enrichment of dietary fiber-degrading bacteria. In addition, in this study, both COS1 and COS2 supplementation enriched the pathway of L-ornithine biosynthesis (GLUTORN-PWY). L-ornithine is an amino acid in the liver responsible for the urea cycle, which can convert toxic ammonia into urea, thereby protecting the health of the viscera (64). Therefore, we hypothesized that COS1 supplementation can increase the degradation efficiency of dietary fiber in feed and improve the intestinal immune of blue foxes.

In this study, the concentration of Mycoplasma in the basal diet was negatively correlated with the intestinal barrier index and positively correlated with the imbalance of inflammation. The enrichment of Mycoplasma is a manifestation of intestinal flora disorder (65). Importantly, *L.agilis* and *L.murinus* enriched in COS1 group and showed a strong positive relationship with intestinal barrier indexes and the balance of inflammatory factors. Intestinal endogenous Lactobacillus is generally distributed on the surface of epithelial cells (66). Studies have shown that 84 and 53 carbohydrate-related enzymes, including helper activity (AAs), glycosyltransferase (GTs), carbohydrate esterase (CEs), and glycoside hydrolase (GH), have been annotated by *L. agilis* and *L. murinus* as potential probiotics. They all showed positive effects on intestinal protection in colitis mice (67, 68), which responded to the enrichment of carbohydrate catabolic pathways in COS1 group as predicted by microbial function. Within 2 weeks of caloric restriction diet, lactic acid bacteria microbial community dominated by *L. murinus_147* rapidly enriched in mice, and this strain reduced intestinal permeability and the level of systemic inflammatory markers, such as TNF-*α* (69), similar to the phenomenon in this study. Further studies have shown that *L. murinus* plays an important role in promoting Treg cell development, inhibiting the development of colitis in mice, and maintaining intestinal immune homeostasis (70). In addition, extracellular vesicles secreted by L. murinus contribute to the polarization of M2 macrophages and the secretion of anti-inflammatory cytokine IL-10 (71). In this study, Succivibrio and Megasphaera enriched in COS2 group also had a certain positive relationship with intestinal barrier and inflammation balance of blue foxes. Succivibrio specializes in the digestion of complex carbohydrates and is a major propionate producer, which is related to feed efficiency (72). Megasphaera, as a probiotic, can ferment lactic acid and mainly produce propionate and valerate, which is related to the improvement of production performance of ruminants (73, 74). The above data explained why the overall supplementation effect of COS1 was better than that of COS2, which may be due to the cascade reaction of intestinal bacteria in response to oligosaccharides with different molecular weights.

In conclusion, dietary COS can improve intestinal morphology and barrier function of growing blue foxes by improving inflammation and immune levels. Our study confirms the strong prebiotic properties of COS in blue fox breeding. The change of COS on the enrichment of different flora in cecum may be the main reason for promoting intestinal health of blue foxes.

## Data Availability

The datasets presented in this study can be found in online repositories. The names of the repository/repositories and accession number(s) can be found below: https://www.ncbi.nlm.nih.gov/, PRJNA1066055.

## Supplementary material

Supplementary Figure 1 Differential enrichment analysis of dietary COS supplementation on phyla level of cecal microflora in growing blue foxes: BD vs COS1.

Supplementary Figure 2 Differential enrichment analysis of dietary COS supplementation on phyla level of cecal microflora in growing blue foxes: COS1 vs COS2.
