# Supplementary figures and images for: Chitooligosaccharides improves intestinal mucosal immunity and intestinal microbiota in blue foxes
Source: Front Immunol. 2024 Nov 19;15:1506991. doi: 10.3389/fimmu.2024.1506991 (PMC11611864; doi:10.3389/fimmu.2024.1506991)

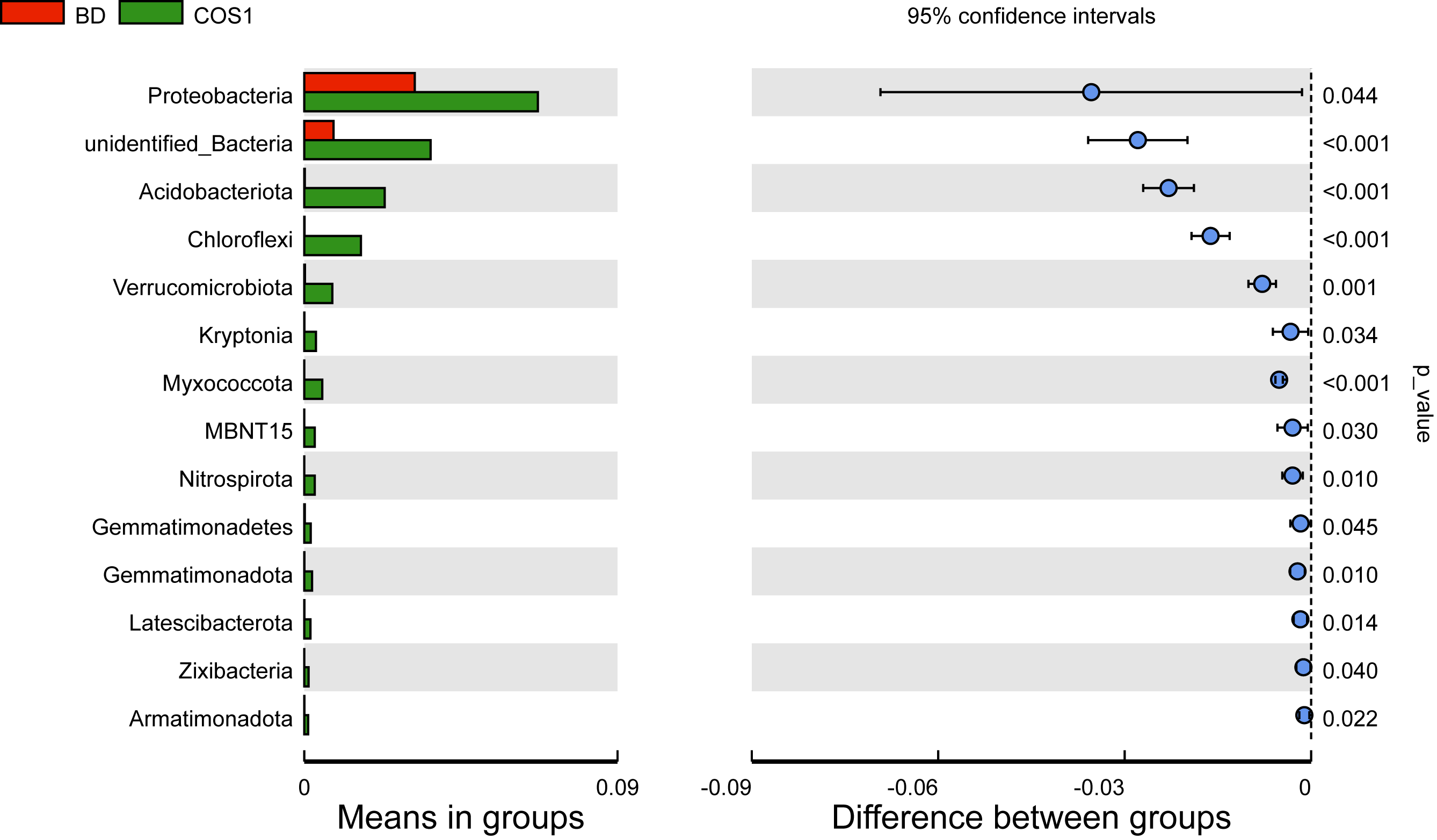

Supplement: Supplementary Figure 1 — Differential enrichment analysis of dietary COS supplementation on phyla level of cecal microflora in growing blue foxes: BD vs COS1. [file Image1.tif]

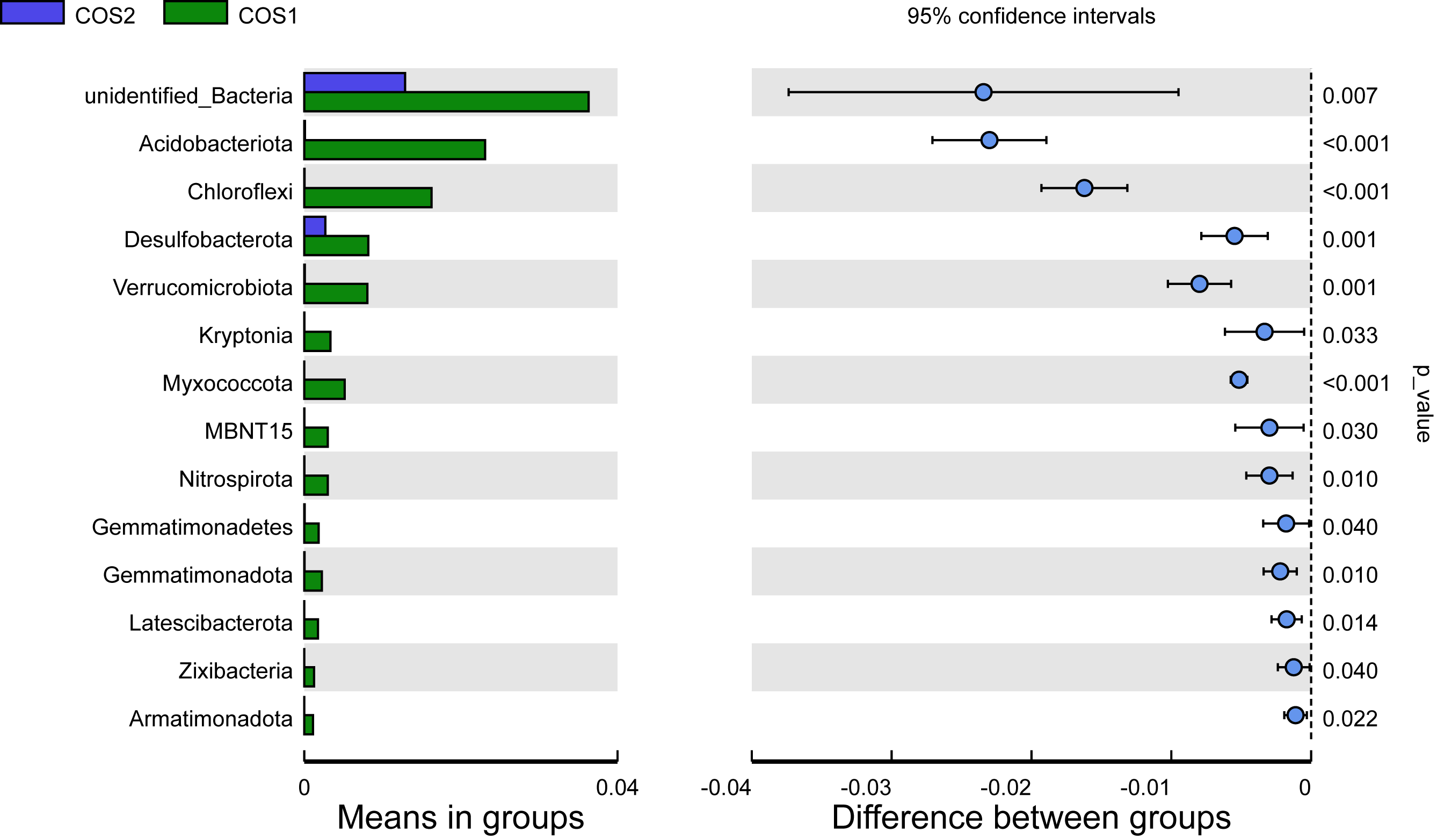

Supplement: Supplementary Figure 2 — Differential enrichment analysis of dietary COS supplementation on phyla level of cecal microflora in growing blue foxes: COS1 vs COS2. [file Image2.tif]
